# Mammalian Neuropeptides as Modulators of Microbial Infections: Their Dual Role in Defense versus Virulence and Pathogenesis

**DOI:** 10.3390/ijms22073658

**Published:** 2021-04-01

**Authors:** Daria Augustyniak, Eliza Kramarska, Paweł Mackiewicz, Magdalena Orczyk-Pawiłowicz, Fionnuala T. Lundy

**Affiliations:** 1 Department of Pathogen Biology and Immunology, Faculty of Biology, University of Wroclaw, 51-148 Wroclaw, Poland; eliza.kramarska@gmail.com; 2 Institute of Biostructures and Bioimaging, Consiglio Nazionale delle Ricerche, 80134 Napoli, Italy; 3 Department of Bioinformatics and Genomics, Faculty of Biotechnology, University of Wroclaw, 50-383 Wroclaw, Poland; pamac@smorfland.uni.wroc.pl; 4 Department of Chemistry and Immunochemistry, Wroclaw Medical University, 50-369 Wroclaw, Poland; magdalena.orczyk-pawilowicz@umed.wroc.pl; 5 Wellcome-Wolfson Institute for Experimental Medicine, School of Medicine, Dentistry and Biomedical Sciences, Queen’s University Belfast, Belfast BT9 7BL, UK; F.Lundy@qub.ac.uk

**Keywords:** neuropeptides, bacterial infections, fungal infections, protozoan infections, defense, pathogenesis, virulence, adhesion, invasion, antimicrobial activity

## Abstract

The regulation of infection and inflammation by a variety of host peptides may represent an evolutionary failsafe in terms of functional degeneracy and it emphasizes the significance of host defense in survival. Neuropeptides have been demonstrated to have similar antimicrobial activities to conventional antimicrobial peptides with broad-spectrum action against a variety of microorganisms. Neuropeptides display indirect anti-infective capacity via enhancement of the host’s innate and adaptive immune defense mechanisms. However, more recently concerns have been raised that some neuropeptides may have the potential to augment microbial virulence. In this review we discuss the dual role of neuropeptides, perceived as a double-edged sword, with antimicrobial activity against bacteria, fungi, and protozoa but also capable of enhancing virulence and pathogenicity. We review the different ways by which neuropeptides modulate crucial stages of microbial pathogenesis such as adhesion, biofilm formation, invasion, intracellular lifestyle, dissemination, etc., including their anti-infective properties but also detrimental effects. Finally, we provide an overview of the efficacy and therapeutic potential of neuropeptides in murine models of infectious diseases and outline the intrinsic host factors as well as factors related to pathogen adaptation that may influence efficacy.

## 1. Introduction

Neuropeptides are a large group of peptides that play a key role in the dialogue between the central nervous system (CNS), peripheral nervous system (PNS), and the immune system. Thus, they can be perceived not only as neurotransmitters but also as hormones or effector compounds that interact with the immune system. Neuropeptides are found in *Homo sapiens* and almost all animal phyla. Since the first discovery of Substance P (SP) by von Euler and Gaddum in 1931, approximately 100 different human neuropeptides and more than 80 genes encoding for neuropeptides in the *H. sapiens* genome have been described [1,2]. Neuropeptides are generally described as macromolecular peptides that contain between 3 and 100 amino acids in their active forms and act via G protein-coupled receptors (GPCR) [3,4,5]. It is acknowledged that neuropeptides have characteristics that are common to peptides, including post translational processing, which may influence their biological activity. Furthermore, depending on receptor expression, they can trigger cell activation at multiple locations, which in the case of neuropeptides may involve both CNS and PNS sites [2,6,7]. Neuropeptides are primarily synthesized in neuronal and glial cells [4,8]. Nevertheless, some neuropeptides are produced by non-neuronal cells, including cells of the immune system. Immune cells such as B- and T-lymphocytes, monocytes, macrophages, dendritic and mast cells as well as polymorphonuclear leukocytes have all been reported to produce these macromolecules [9,10,11]. Fibroblasts have also been shown to synthesize selected neuropeptides and neuropeptide receptors [12,13]. Since immune cells participate in a bidirectional conversation with the nervous system and other stromal cells, they act not only as producers of neuropeptides but also detect neuropeptides by neuropeptide-specific GPCRs, or by mannose receptors via a so-called “non-specific” receptor mechanism. Likewise, receptor-independent cellular activation is also possible [11,14]. Considering the interaction of neuropeptides with their specific GPCR receptors, most neuropeptides can induce modulatory actions in the cell cytoplasm via GPCR-linked pathways. The quantity of neuropeptide that is required to trigger such action is significantly lower than required for other neuro-signaling molecules [4,15]. This is perhaps related to the affinity and bond strength between the neuropeptide and the GPCR, which appears to be enhanced compared with the classical neurotransmitter-receptor linkage. In addition to their interaction with their cognate receptors, neuropeptides have also been reported to trigger ionotropic reactions [16]. 

Beyond the traditional role of neuropeptides as neurotransmitters in the central and peripheral neural systems, they have also antimicrobial activity contributing to the formation of local barriers of defense against a variety of pathogens. However, recent data also indicate that neuropeptides can contribute to modulating vulnerability to infection. Thus, in addition to their anti-infective action, some neuropeptides may have a completely different, unfavorable role to play in augmenting bacterial virulence. In this review, we will summarize current data on the dual modulatory role of neuropeptides in infectious diseases caused by bacteria, fungi, and protozoan parasites, including their impact on virulence, pathogenesis, and possibility of resistance development. We also critically discuss attempts to date to employ neuropeptides in animal models of infections, together with future perspectives for these important molecules. This review mainly focuses on the following neuropeptides: substance P (SP), neuropeptide Y (NPY), calcitonin gene-related peptide (CGRP), vasoactive intestinal peptide (VIP), pituitary andenylate cyclase-activating polypetide (PACAP), adrenomedullin (ADM), and somatostatin (SST). We also discuss the peptide hormones such as α-melanocyte stimulating hormone (α-MSH), atrial natriuretic peptide (ANP), C-type natriuretic peptide (CNP), and corticotropin-releasing hormone (CRH) as well as dynorphin as examples of opioid peptides.

## 2. Neuropeptides as Direct and Indirect Anti-Microbial Factors

The antimicrobial properties of human neuropeptides such as SP, NPY, CGRP, VIP, ADM, and α-MSH against a variety of microorganisms are widely documented. Their direct antimicrobial activities have been confirmed to date against Gram-negative (*Escherichia coli*, *Pseudomonas aeruginosa*, *Haemophilus influenzae*, *Moraxella catarrhalis*, *Aeromonas caviae*) and Gram-positive bacteria (*Staphylococcus aureus*, *Enterococcus faecalis*, *Streptococcus mutans*, *Nocardia brasiliensis*), fungi (*Candida albicans*, *Candida tropicalis*, *Candida krusei*, *Candidia utilis*, *Cryptococcus neoformans*, *Arthroderma simii*), and protozoan parasites (*Trypanosoma brucei*, *Leishmania major*) [11,17,18]. Interestingly, some highly proteolytic oral anaerobic bacteria such as *Porphyromonas gingivalis* and *Prevotella* spp have been reported to be resistant to the direct action of CGRP and ADM [19,20]. The direct action of neuropeptides encompasses both microbicidal and anti-virulent effects. The microbicidal action of neuropeptides is generally attributed to targeting the microbial membrane and causing either discrete pore formation or more extensive detergent-like disruption of the membrane. In either case, the effect is rapid, with leakage of microbial cytoplasmic contents, leading to cell death within minutes [21]. Less common, non-membrane-disruptive mechanisms of direct activity and killing have also been reported, including (i) Abnormal septum formation (scaffolding for peptidoglycan) during cell division by *S. aureus* in the presence of ADM [22] and (ii) ADM- and VIP-mediated disruption of intracellular endosome-lysosomal vesicles, resulting in metabolic failure in *T. brucei* [23]. Of note, the anti-virulent action of neuropeptides may be exemplified by the anti-proliferative (bacteriostatic) activity of somatostatin against *H. pylori* [24]. 

Considering membrane-disruptive mechanisms, an advantage of neuropeptides, like other AMPs, is their efficacy against both metabolically active and metabolically repressed microbial cells. Conversely, their significant drawback is generally rather weak microbicidal effects at physiological doses. Readers are referred to previous reviews by Augustyniak et al. and Sanz et al. [11,25] for deeper insights into the mechanisms of direct antimicrobial activities of neuropeptides. 

However, in addition to direct action, neuropeptides may also display antimicrobial activity via indirect immunomodulatory effects on the innate and adaptive immune systems during the course of infection [6,26]. This humoral and cell-mediated immune modulation may be either stimulatory or inhibitory, implicating that neuropeptides have a dual role. For immunomodulating purposes, neuropeptides exploit specific receptors and also, paradoxically, utilize alternative non-cognate receptors or non-receptor-mediated mechanisms. During certain receptor–neuropeptide interactions, dual biological effects, such as for example stimulation versus inhibition of critical phagocytic events have previously been documented [11]. The stimulating potential of neuropeptides is essentially based on the intensification of mechanisms of innate defense. Modulation of critical phagocytic or mast cell function as well as triggering of proinflammatory mediators is worth mentioning in this context [6,27,28]. Conversely, a common example of inhibitory action is neuropeptide-mediated inhibition of pathogen-induced inflammation or reducing endotoxin-induced inflammatory responses. For more comprehensive information on these, we refer the reader to the previous reports [10,21,29]. 

It is important to note that the direct and indirect antimicrobial activities of neuropeptides presented above, are derived from in vitro studies, conducted under strictly controlled laboratory conditions, and therefore may not necessarily reflect the in vivo efficacy of the neuropeptides.

## 3. Neuropeptides as Direct Potentiators or Amplifiers of Microbial Virulence

**Figure 1:**
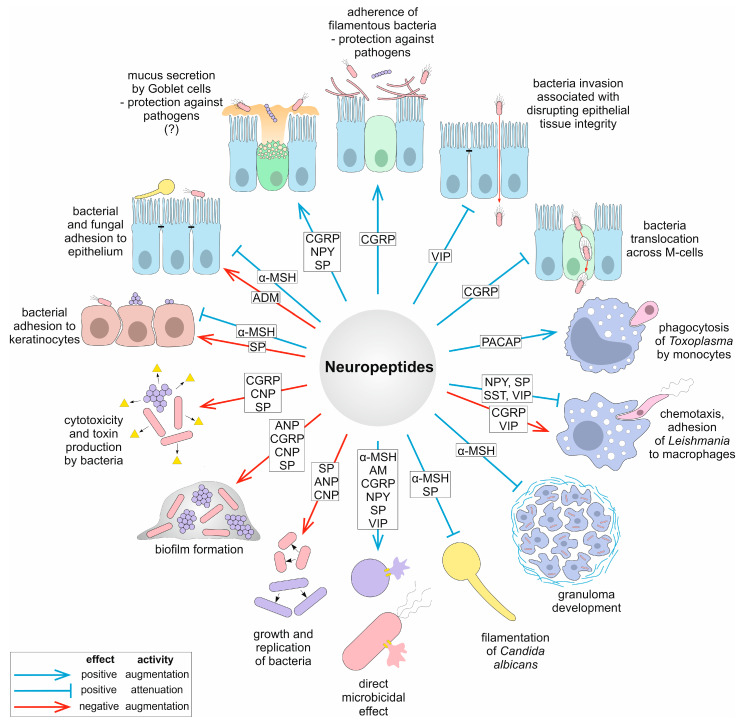
Overview of mechanisms of pathogenesis and virulence traits modulated by neuropeptides. The question mark indicates that the given mechanism is putative.

Virulence is defined as the ability of a pathogen to cause disease. Virulence factors are secretory, membrane-associated, or cytosolic molecules produced by bacteria, fungi, and protozoa that enable them to colonize the host at a cellular level. These factors include, among others, adhesins, toxins, enzymes, as well as factors involved in biofilm formation. Despite the role of neuropeptides in the host’s intricate defense mechanisms to control microbial invasion [11,25], these pleiotropic peptides may under certain circumstances be considered to have virulence-enhancing features [30]. The potential virulence-enhancing roles of neuropeptides can be summarized by their ability to (1) increase microbial growth; (2) increase thickness and density of microbial biofilm; (3) increase exotoxin production or bacterial cytotoxicity; (4) interfere with quorum-sensing that may control expression of virulence factors. Previous reports demonstrating the detrimental effects of neuropeptides on virulence traits are summarized in Table 1 and Figure 1 and discussed in further detail below.

### 3.1. Influence on Microbial Growth and Virulence

Some neuropeptides may exert virulence-enhancing action while inducing cellular stress in host microenvironment. Good examples include epinephrine (EPI), norepinephrine (NE), and SP that can trigger commensal-to-pathogen transition of *Mennheimia haemolytica* in the nasopharyngeal milieu. This Gram-negative bacterium is an important constituent within commensal biofilm communities in the bovine nasopharynx and can, under certain circumstances, play a key role in bovine respiratory disease. EPI, NE, and SP have been shown to significantly increase the growth and replication of *M. haemolytica* but also to trigger its transition from natural biofilm-former to pulmonary planktonic pathogen. The higher rate of cell division is generally thought to be connected to changes in bacterial virulence including production of the important cytotoxic virulence factor leukotoxin Lkt, which causes lysis of erythrocytes and induces apoptosis in macrophages [31,32]. The typically harmless bacterium *M. haemolytica* is therefore released from the biofilm community in the nasopharynx and spreads to a new site of infection in the lungs, causing bronchopneumonia [33]. In contrast, SP was shown not to affect the growth of selected Gram-positive (*Bacillus cereus*, *S. aureus*, and *Staphylococcus epidermidis*) and Gram-negative (*Pseudomonas fluorescens*) bacteria, although it was reported to enhance the virulence of *Bacillus* and *Staphylococci* [34], Table 1. Interestingly, enhancing fungal virulence has previously been exploited for human advantage. A mycoinsecticidal strategy has been used in the treatment and killing or fire ants, which are destructive insects that can easily spread to new ecosystems. It was shown that the expression of a fire ant pyrokinin β neuropeptide in the entomopathogenic fungus *Beauveria bassiana* strain improved the virulence of this fungus. As a result, the more virulent mycoinsecticidal fungal strain was able to kill fire ants and alter their behavior [35].

### 3.2. Influence on Biofilm Formation and Quorum Sensing

To date, the most intensely studied virulence process modulated by neuropeptides is biofilm formation [30] (Table 1, Figure 1). Biofilms facilitate colonization of the host by a wide range of microorganisms and constitute a barrier designed to counteract host defense and therapeutic intervention. Microorganisms within biofilms favor the survival of the entire community, which is achieved through subsequent stages of biofilm formation such as adherence, aggregation, and matrix formation, maturation, and dispersion (release of microcolonies) [36]. It has been shown that Gram-positive bacteria such as *B. cereus*, which causes food poisoning, and the opportunistic bacterium *S. epidermidis*, which inhabits the skin, develop significantly thicker biofilms after exposure to SP [37,38,39]. The population density in a biofilm is controlled through a complex cell-to-cell signaling mechanism known as quorum sensing (QS) [40,41]. During QS, bacteria produce, detect, and respond to extracellular signaling molecules called autoinducers and processes controlled by QS include, but are not limited to, biofilm formation and virulence factor secretion [42,43]. SP and also CGRP hypothetically may affect *S. epiderimidis* factors responsible for QS signaling which in turn affect biofilm formation. Following a mechanism potentially close to that of bacterial QS factors, but not yet identified precisely, for a factor known as ribosomal elongation factor thermo-unstable (Ef-Tu), which is engaged in translation, it has been shown that after binding to SP or when chaperone DnaK binds to CGRP, bacterial response such as increased adhesion and biofilm formation should be triggered [34].

**Table 1:** Overview of detrimental effects of neuropeptides on virulence traits.

Bacteria	NP	Type of Virulence Modulation	Field of Action	Ref.
*M. haemolytica*	SP	Dispersion from biofilm ↑ Growth and replication ↑ Lkt protein production ↑	Adherence, biofilm formation, spreading Direct damage	[31]
*B. cereus*	SP	Biofilm thickness ↑ Cytotoxic effect ↑	Host immune response evasion, adherence Direct damage	[37]
*S. epidermidis*	SP	Biofilm thickness and density ↑	Host immune response evasion, adherence	[38]
*S. aureus*	SP	SEC2 superantigen production ↑	Acute host immune response	[38]
*S. pneumoniae*	CRH	Capsule thickness ↑ Growth ↑ Pneumolysin Ply production ↑ pavA production ↑	Adherence, host immune response evasion Proliferation tempo Direct damage Adherence, invasion	[44,45]
*S. epidermidis*	CGRP	Cytotoxic effect ↑ Cationic potential of surface ↑ IsaB production ↑	Direct damage Adherence Host immune response evasion	[46]
*C. acnes*	ANP, CNP	Growth ↑ Biofilm formation ↑	Proliferation tempo Host immune response evasion, adherence	[47]
*S. epidermidis*	ANP, CNP	Biofilm formation ↑	Host immune response evasion, adherence	[48]
*P. aeruginosa*	Dynorphin	HHQ and PQS molecules production ↑ pyocyanin production ↑ PA-I lectin/adhesin ↑	Quorum sensing Direct damage, competition with other microorganisms Host immune response evasion Adherence	[49]

**↑**—increase; NP—neuropeptide; HHQ—4−hydroxy-2-heptylquinoline; PQS—3,4-dihydroxy-2-heptylquinoline; SEC2—enterotoxin C2.

### 3.3. Influence on Microbial Toxin Release and Strain Cytotoxicity

Toxin production represents an efficient way to alter specific functions of target cells, enabling pathogenic effects. After exposure to CGRP, *S. aureus* increased production of enterotoxin C2 (SEC2) [46]. This virulence factor is a member of the super-antigen group, which possesses the ability to activate high numbers of host T-cells by crosslinking with Major Histocompatibility Class II (MHCII) molecules, thereby inducing profound and rapid cell activation, which can result in a generation of a cascade of cytokines, leading to toxic shock syndrome [50,51]. Another sensory neuropeptide, SP, has also been shown to affect bacterial cytotoxicity. This effect was confirmed for *B. cereus* against a cell line of human keratinocytes [37] as well as for *S. aureus* and *S. epidermidis* against a reconstructed human epidermis model [38,46]. SP also stimulate aggregation, hydrophobicity, lactic acid and tyramine production in *E. faecalis*, an important nosocomial pathogen causing severe urinary tract infections, surgical wound infections, bacteraemia, and bacterial endocarditis. SP was also shown to accelerate the cytotoxicity and translocation of this bacterium during infection of intestinal Caco-2/TC7 cells [52]. Another interesting example in this matter is CNP that modulates QS molecule and exotoxin A production in *P. aeruginosa* through a cyclic nucleotide-dependent sensor system. As a result, a toxin-dependent capacity of *P. aeruginosa* to kill nematode *Caenorhabditis elegans* was increased markedly. This activity was related to activation of bacterial regulators of virulence such as Vfr and PtxR, as well as the virulence activation cascade involved in production of acylhomoserine lactone, hydrogen cyanide, and aforementioned exotoxin [53].

### 3.4. Potential Influence on Immune Evasion

Pathogens utilize a vast array of sophisticated strategies to evade the host immune response. Generally, pathogens actively block or degrade immune components as well as modulate the cellular machinery of infected cells on various levels to allow cellular invasion and then transmission. Surface components of pathogens often play an important passive role in immune evasion. One of such components in *Streptococcus pneumoniae* is a capsule, a polysaccharide layer that surrounds a cell. After exposure of *S. pneumoniae* to CRH, the thickness of the capsule was markedly enhanced. This was evident for serotypes 1, 3, 19A and 23F, among the major causes of invasive pneumococcal disease. Furthermore, CRH-treated bacteria presented 3-fold greater resistivity for penicillin/streptomycin cocktail compared to control [45]. Since capsules generally act as an armor, that prevents complement-dependent cell lysis and C3b- or IgG-dependent opsonization that leads to phagocytosis enhancement [54], it is reasonable to suggest that CRH may make it harder for the phagocytic cell to recognize and phagocytize this encapsulated pathogen. Likewise, *S. aureus* immunodominant surface antigen B (IsaB) and precursor protein were shown to be increased during CGRP exposure in vitro [46]. The IsaB protein appears to be expressed during infection but not colonization and is increased in phagocytosis [55]. Since IsaB diminishes autophagic flux, to promote bacterial survival and host transmission, allowing Methicillin-Resistant *S. aureus* (MRSA) to evade host degradation [56], it is reasonable to suggest that a CGRP-dependent increase in expression of this surface antigen may favor bacterial survival and dissemination. 

### 3.5. Pathogen Sensing of Neuropeptides 

During the course of an infection, microorganisms depend on their ability to sense and respond to a myriad of environmental host factors and the activation of specific virulence traits that are needed to establish an infection successfully. Because the expression and/or activation of these virulence traits is metabolically expensive, it should occur only at sites that are appropriate for colonization [57]. Assuming that various antimicrobials, including neuropeptides, significantly alter the metabolic state of microorganisms, it seems that the microbes per se may also affect their intrinsic susceptibility to neuropeptides via moonlighting proteins. Indeed, bacteria and other pathogens have the ability to sense neuropeptides via highly conserved proteins, termed moonlighting proteins, which exhibit more than one unrelated function within the cell. Since the novel biological function of the moonlighting protein is usually connected with a change in its localization, moonlighting proteins often perform their canonical and moonlighting functions in separate cell compartments. Pathogens commonly use moonlighting cytosolic proteins engaged essentially in cellular processes (i.e., glycolysis, protein synthesis, chaperone activity, and nucleic acid stability) on their cell surface for forming and maintaining interactions with the host epithelial cells, mucus, and extracellular matrix (ECM) components [58,59]. A well-characterized example is the enzyme glyceraldehyde-3-phosphate dehydrogenase (GAPDH) of *Streptococcus pyogenes* or *S. aureus*. This enzyme is involved in adherence and internalization and thus directly serves as a virulence determinant for adhesion [60,61]. There is also an array of moonlighting proteins, with adhesive properties, in the yeast *C. albicans*, including enolase (ENO1), phosphoglycerate mutase 1 (GPM1), Ssa1 chaperoning, and GAPDH [62,63,64]. Another unique function of microbial moonlighting proteins involves the evasion of the innate immune system, in particular by binding of serum complement components or complement regulators (observed for the moonlighting proteins dihydrolipoamide dehydrogenase LpD, factor EfTu, GAPDH) [65,66,67]. Moonlighting proteins have also been shown to bind and potentially neutralize host antibodies (LpD, GroEL chaperone) [68]. Scientists have long been puzzled by the question of whether, analogous to other components of the humoral immune response, neuropeptides (which could be considered as part of this response) could also be sensed by moonlighting proteins. To our knowledge, this has only been confirmed in a few cases. One of them is the aforementioned bacterial environmental sensor Ef-Tu, which is as a molecule involved in the recognition of SP in *S. aureus*, *S. epidermidis*, and *B. cereus*. Interaction between Ef-Tu and SP led to increased bacterial adhesion potential and biofilm formation in a keratinocyte model of infection [38,39]. The same group of scientists identified DnaK chaperone as the *S. epidermidis* CGRP-binding protein. Following exposure to CGRP, the adherence of *S. epidermidis* to keratinocytes, as well as the cytotoxicity to these cells, was increased, whereas its internalization and biofilm formation activity was reduced. Of note, the CGRP-mediated increase in virulence was not observed for *S. aureus* [46], indicating therefore that even in closely related bacteria, the effects of the same neuropeptide can be quite different. Another well-documented example is the *P. aeruginosa* kinase (ParS) involved in the sensing machinery to defend against the host in response to the opioid neuropeptide dynorphin [69]. Host dynorphins, released from the intestinal mucosa, can activate quorum sensing quinolone signaling in *P. aeruginosa* using transcriptional regulator MvfR, and enhance the virulence of *P. aeruginosa* against *Lactobacillus* spp. and nematode *C. elegans*. These data demonstrate that *P. aeruginosa* can intercept opioid compounds released during host stress and integrate them into core elements of quorum sensing circuitry leading to enhanced virulence [49]. In other cases, the involvement of bacteria in recognizing certain neural peptides has been documented, but without identifying the bacterial molecular targets engaged in this recognition [30]. The lack of other experimentally documented examples of neuropeptide- or antimicrobial peptide-binding by bacterial moonlighting proteins seems to indicate that this virulence trait is unlikely to be generally preferred by bacterial pathogens, excluding those already mentioned above. It may be assumed that from a pathogen’s perspective, the virulence-enhancing benefits that the bacterium derives from using moonlighting proteins for adhesion or evasion of immune response far outweigh the other benefits following neuropeptide binding by these proteins. In this aspect, one can speculate that the direct sensing of neuropeptides by bacteria appears to be a dead end from evolutionary perspective.

## 4. Neuropeptides Modulate Crucial Stages of Microbial Pathogenesis

The ability of a pathogen to infect is called its pathogenicity. Microorganisms express their pathogenicity by means of their virulence, a term that refers to the relative, quantitative degree of pathogenicity [70,71]. Different microbial pathogens use various common strategies to cause infection and disease. These strategies enable them to attach, invade, and disseminate in the host, as well as acquire nutrients for their growth and multiplication within the host. In vivo, a pathogen must also evade continuous attacks from the host immune response and actively survive host defense mechanisms. Some pathogens from the lower Eukaryota have developed other mechanisms of pathogenesis such as yeast-to-hyphae transition (dimorphism); a mechanism exhibited by pathogenic yeasts such as *C. albicans* [72]. For protozoan parasites, it involves several different stages during their life cycle and a remarkable ability to manipulate host immunity [73,74]. Numerous pathogenic effects can be attenuated or enhanced in the presence of neuropeptides, placing these molecules amongst the important modulators of microbial pathogenesis. The involvement of neuropeptides as modulators of crucial stages of microbial pathogenesis has allowed the host to develop intricate and overlapping mechanisms to control microbial invasion. To date, the most intensely studied stages regulated by neuropeptides involve their interplay with microbial colonization and adhesion, invasion, intracellular lifestyle, and growth or multiplication (Figure 1). The dual role in exerting either beneficial or detrimental effects is outlined below with recent examples.

### 4.1. Impact of Neuropeptides on Microbial Colonization and Adhesion

The ability of microbial pathogens to successfully colonize the host relies initially on their ability to break down physical and chemical barriers. Following this, pathogens must be able to attach to host cells. Depending on the pathogen, attachment may be mediated by surface-associated protein structures such as fimbriae or pili, numerous surface proteins, and the capsule (if present). Since adhesion is the first step in the process of colonization and subsequent infection, inhibition of adhesion should be expected to limit the pathogenicity of at least some microorganisms. Evidence of a negative role for neuropeptides in bacterial adhesion has come from several approaches, including studies on cutaneous pathogens. It has been shown that SP increases adhesion of Gram-positive *S. aureus* and *S. epidermidis* to skin keratinocytes several times over control. Surprisingly, although adhesive potential was increased, invasive activities expressed by internalization of staphylococci by these cells were unchanged during experimental conditions [38]. As documented elsewhere, keratinocytes generally had a much lower uptake of *S. aureus* than, for example, the endothelial or epithelial cells [75]. Besides staphylococci, SP also increased the adhesion and invasive potential of another cutaneous bacterium, *P. fluorescence* in human keratinocytes [76]. Similarly, the negative effect of the neuropeptide ADM on colonization has been documented for invasive, facultative intracellular bacterium *Helicobacter pylori*, a leading mucosal pathogen of chronic gastritis and other digestive system diseases which colonize the stomach of its host [77]. In this case, ADM expression was positively correlated with *H. pylori* colonization in gastric mucosa [78]. Generally, the adhesion of *H. pylori* to gastric mucosa epithelial cells is mediated by outer membrane proteins and the colonization is expedited in a favorable inflammatory environment. This is facilitated by the delivery of *H. pylori* CagA effector protein, encoded by genes located within pathogenicity islands, to the mucosal epithelium, via interaction of CagA with integrin β1 in a type IV secretion (T4SS)-dependent manner. Another model for T4SS-dependent CagA translocation involves binding of pilus-exposed CagA to a host membrane phospholipid, phosphatidylserine. Both models permit CagA translocation in host epithelial target cells [79,80]. Apparently, ADM released from the gastric epithelium via *cag*A-dependent activation of PI3K-AKT signaling pathway, initiated inappropriate immune response and inflammation, creating a favorable environment for pathogen survival and colonization. This was partially due to direct induction of T cell response as well as indirect activation of T cells via IL-12, produced by ADM-activated macrophages. In both cases, Th1 lymphocytes release IFN-γ that exerts a pro-inflammatory effect within the gastric microenvironment, thus contributing to gastritis during *H. pylori* infection. It is worth adding that ADM levels have been shown to be increased in the gastric mucosa of *H. pylori*-infected patients and mice [78]. Interestingly, when the external conditions become unfavorable for bacteria to establish successful colonization, adhered *H. pylori* invade epithelial cells and multiply within double-layer membrane vesicles present either on the cell membrane or in the cell cytoplasm. Once the external environment conditions improve, *H. pylori* can be released from host cells for recolonization. In this way, bacterial numbers are maintained through a dynamic process involving invasion, proliferation, apoptosis, and release [79]. 

In contrast to the aforementioned examples of adverse effects of some neuropeptides, which are based on increasing adhesion, the effect of α-MSH on the initial stages of infections caused by *S. aureus* may be positive for the host. In the case of α-MSH, its antibacterial effects are connected with inhibition of adhesion and penetration during the early stages of infection. For example, in human keratinocytes α-MSH down-regulates β-1 integrins and heat shock surface protein 70, both of which are essential molecules for the invasion of keratinocytes by *S. aureus* [81]. 

Furthermore, more complicated effects of neuropeptides have been reported for obligatory intracellular protozoan parasites from *Leishmania* genus. *Leishmania* exhibits two main distinct developmental stages: (i) the free-living flagellated and invasive promastigote and (ii) the obligate intracellular aflagellated amastigotes living in phagolysosomes of macrophages [82]. For this pathogen, which causes mucocutaneous leishmaniasis, active migration followed by adherence and invasion into skin macrophages are key steps for initiation of its successful replication. However, these resident cells are also the major effector cells to eliminate the parasite. This duality in pathogen-cell response depends on which subset of macrophages is activated. It therefore may lead to different disease outcomes. More precisely, macrophages can be activated by divergent signals into functionally distinct subsets such as *Leishmania*-killing M1 and *Leishmania*-tolerating M2 [83]. Interestingly, by acting as pathogen chemorepellents or chemoattractants, certain neuropeptides at physiological concentrations can either block chemotaxis followed by adhesion and thus prevent parasite invasion into macrophages or alternatively, attract the parasite. Neuropeptides released from the autonomic nervous system, such as NPY and VIP, were shown to act as direct chemorepellents against *Leishmania brasiliensis* flagellated promastigotes. In contrast, the chemoattractive sensory neuropeptide SP was shown to cause decreased promastigote adherence to macrophages, thus indicating that despite inducing parasite migration, SP impairs parasite adhesion potency [84]. The reasons for these discrepancies require further research. Likewise, *Leishmania major* promastigote-induced macrophage migration was shown to be modulated in a dual way by sensory and autonomic neuropeptides. In this case, physiological concentrations of SST, SP, and NPY inhibited *L. major*-induced chemotaxis of phagocytic macrophages, whereas VIP and CGRP stimulated the chemotactic activity of these cells [85]. Interestingly, the potency of neuropeptides to modulate chemotaxis (initial step in phagocytosis) did not always correlate with later stages of phagocytosis such as ingestion and killing. Accordingly, the autonomic NPY and VIP have been shown to suppress phagocytic and leishmanicidal capacities of macrophages, whereas inhibition of phagocytosis by sensory SP and CGRP was accompanied by an enhancement of parasite killing [86]. Since macrophages constitute a reservoir for these obligate intracellular parasites and are crucial in disease progression, by preventing *Leishmania* from accessing these cells, certain neuropeptides seem to exert potent anti-adhesive properties, contributing to protection against this pathogen. In turn, certain other neuropeptides are useful in enhancement of leishmanicidal properties of infected cells. Another example of the anti-adhesive action of neuropeptides is α-MSH- and galanin message-associated peptide (GMAP)-mediated inhibition of germ-tube formation in *C. albicans*, which in turn limits yeast filamentation [87,88]. Furthermore, during our recent studies, we have observed a clear effect of SP in decreasing both the number and length of hyphal filaments in the pathogenic yeast *C. albicans* in the presence of serum and fluconazole (unpublished data). Since filamentous hyphal forms of this pathogenic yeast adhere more robustly and efficiently to host cells than yeast cells, and hyphae are considered dominant invasive forms of the fungus [89,90], it is reasonable to suggest that α-MSH, GMAP, and potentially also SP may have a role in blocking *Candida* adhesion.

### 4.2. Impact of Neuropeptides on Mucus Secretion 

The adherence of pathogens to the epithelium of the respiratory, gastrointestinal, and urogenital tracts prevents their mechanical clearing. Fortunately, adherence is usually impaired in the presence of a physiologically produced mucus—a thick and viscous colloid hydrogel. The major sources of mucus are the numerous mucous cells located in the surface epithelium (called goblet cells) and in submucosal glands [91,92]. The ability of normal mucus to suppress pathogen growth depends on synergistic actions between its complex components (water, electrolytes, mucins) with antimicrobial humoral and cellular immune constituents. The protective role of the gel-forming, glycoprotein mucin is to trap bacteria that possess protein and carbohydrate ligands for mucin and block their access to other mucous cells. It has been shown that in normal conditions, neuropeptides such as SP, CGRP, NPY may exert a modulatory role in mucus secretion primarily through stimulation of goblet cells and stimulation of airway submucosal glands [93]. Therefore, by inducing favorable mucus secretion, key neuropeptides may indirectly interfere with microbial colonization. It is worth adding that mucus also plays an important role in the differentiation and behavior of microbial phenotypes of both commensals and pathogens [94].

Knowing the numerous benefits of the protective antibacterial effect of mucus, one cannot forget that mucus obstruction is an important feature of some chronic respiratory diseases including chronic obstructive pulmonary disease (COPD), cystic fibrosis (CF), and asthma. Since mucus is an important defensive mechanism, its increased or defective secretion, together with improper composition, can alter its functionality. Poor mucus clearance can contribute to airway inflammation and persistent bacterial infections. In CF, the defective secretion of mucus by submucosal glands is a consequence of lack of functional CF transmembrane conductance regulator (CFTR) chloride channels. Furthermore, in CF airways, mucus glands form tethered mucus plaques in which various antimicrobials become ineffective [95]. Moreover, conditions within the plaques, such as lower O_2_ tension, contribute to the formation of a mucoid and more resistant phenotype among residing bacterial pathogens such as *P. aeruginosa* [95]. It was demonstrated that CFTR of submucosal glands in CF patients may be abnormally activated in a CGRP-dependent manner. As a consequence, mucus production and proliferation of glandular progenitor cells may occur [96]. Under these circumstances, the aforementioned CGRP-mediated stimulation of mucus secretion will certainly be detrimental. Likewise, chronic bronchitis and asthma exacerbation are associated with elevated SP in sputum and SP-mediated neurogenic inflammation, contributing to airway narrowing [97]. Although the increase in SP levels in mucus is equivocal in COPD [93], it has previously been reported that this neuropeptide may be involved in the pathogenesis of some of the changes observed in COPD, as evidenced by increased SP- and VIP-immunoreactivity reported in epithelium and glands from COPD patients [98].

### 4.3. Impact of Neuropeptides on Tissue Integrity 

Many bacterial pathogens can break epithelial integrity using an array of toxins that target occludin, a protein that plays a crucial role in tight junction structure and contributes to maintaining adequate epithelial permeability. These include toxins that can cause redistribution of occludin, such as *Clostridium difficile* toxin, or EspF of enteropathogenic *E. coli* but also toxins that cause proteolytic degradation of occludin such as *Vibrio cholera* cytotoxin [99,100]. Neuropeptides may help to partially restore epithelial barrier integrity after its disruption caused by enterotoxigenic *E. coli* (ETEC). It has been shown that VIP administered intraperitoneally to piglets may improve epithelial barrier function by preventing the disadvantageous translocation of occludin and other tight junction proteins. This phenomenon was accompanied by a reduced expression of proinflammatory cytokines induced by ETEC, possibly through modulation of Toll-like receptors (TLRs), nuclear factor κB (NF-κB), and mitogen-activated protein kinase (MAPK) pathways. Furthermore, VIP has been shown to upregulate occludin expression in ileum mucosa [101]. Likewise, VIP can stimulate diverse microbial communities associated with increased ecosystem stability in the gastrointestinal tract and resistance to pathogen invasion [102]. In this way, by minimizing bacteria-induced redistribution of tight junction proteins, VIP restores the colonic epithelial barrier, thus contributing to its protection.

### 4.4. Impact of Neuropeptides on the Infectious Inflammatory Response

In addition to disrupting tissue integrity, some pathogens modulate the inflammatory response and neuropeptide release during infection. They often utilize for this purpose evolutionarily conserved and unique for them soluble or surface-associated pathogen-associated molecular patterns (PAMPs). PAMPs of certain bacteria and pathogenic yeasts were shown to directly activate nociceptor TRPV1-positive (transient receptor potential vanilloid 1) neurons through interaction with pattern recognition receptors (PRRs). For example, LPS from *S. enteritidis* can increase levels of dopamine in the brain and neuropeptides such as SP, NPY, VIP, and galanin in cervical lymph nodes [103]. *S. aureus,* a major cause of wound and surgical infections leading to painful abscesses, was shown to stimulate TRPV1-positive neurons by releasing N-formylated peptides, acting on the formyl peptide receptor 1 expressed on nociceptors [104]. In a similar manner, β-glucans from *C. albicans* cell wall directly activate TRPV1-positive neurons by binding to the dectin-1 receptor on nociceptors [105,106]. Other bacterial PAMPs such as peptidoglycan, lipopolysaccharide, flagellin, lipoteichoic acid, as well as the pore-forming toxin alpha-haemolysin can also directly activate TRPV1-postive neurons, through a variety of distinct mechanisms [104,107]. Once activated, this specialized population of pain-sensing (nociceptive) neurons release neuropeptides from peripheral afferent terminals, which in turn act on immune cells, epithelial cells, and the enteric nervous system to maintain homeostasis [108]. Chiu et al. reported that neuropeptides released from nociceptors, upon acute staphylococcal infection, can directly modulate innate immune activation. The most potent of these neuropeptides, namely CGRP, Gal, and SST, suppressed TNF-α release from macrophages, thus contributing to down-regulation of the local inflammatory response [104]. It can therefore be assumed that the direct interplay between staphylococci and nociceptors serves the former in creating a suitable environment for pathogen to survive. Sensory neurons are also engaged in effective anti-fungal responses. For example, *C. albicans* directly affects TRPV1-postive nociceptive sensory neurons, causing release of CGRP. Then neuropeptide drives IL-23 production from dermal CD301b+ dendritic cells. IL-23 elicits IL-17 production from dermal lymphocytes Tγδ, thus inhibiting cutaneous *C. albicans* infection [109]. Therefore, in the skin, sensory neurons following direct sensing of yeast enhance host resistance to this pathogen via secretion of neuropeptide. This eminent cooperation between nervous and immune components provides critical host defense against fungal pathogens during the early stages of skin infection. Sometimes the same sensory neurons may exert opposing effects, which can lead to enhancement and survival of pathogens during infection. This occurs for example during aggressive bacterial subcutaneous infection of connective tissue leading to necrotizing fasciitis. In response to the *S. pneumoniae* toxin, streptolysin S, TRPV1-positive neurons produce pain and release CGRP locally. Local release of CGRP suppresses the recruitment and bactericidal activity of neutrophils. In this way, CGRP released by neurons into infected tissue blocks a pivotal cellular innate response against invasive bacteria [110].

### 4.5. Impact of Neuropeptides on Microbial Invasion 

Once bacteria have successfully colonized their host, some bacterial species will invade the tissues and cells including monocytes or macrophages or epithelial cells whilst others are not capable of intracellular invasion. Extracellular pathogens multiply and colonize the surface of host cells and perhaps break down the barriers of tissue to proliferate and disseminate, but they remain outside of host cells (*S. pyogenes*, *P. aeruginosa, E. coli*). On the other hand, intracellular pathogens may actively penetrate host cells and survive within their environment. Intracellular pathogens are classified as: (i) obligate intracellular pathogens which cannot live outside host cell (certain bacteria: *Chlamydia*, *Rickettsia*, certain parasites: *Toxoplasma* spp.), (ii) facultative intracellular pathogens capable of living and reproducing either inside or outside host cells (*Salmonella enterica* serotype Typhimurium, *Neisseria meningitidis*, *Mycobacterium* spp.), and (iii) non-classical facultative intracellular pathogens, which are internalized by variety of cells and can survive within them (*S. aureus*) [92,111]. There are many infectious diseases where a dual extracellular/intracellular style of infectivity coexists including salmonellosis, tuberculosis, or plague [112]. Divergent intracellular strategies to invade the host can be modulated by certain neuropeptides as discussed below.

#### 4.5.1. Impact of Neuropeptides on Facultative Intracellular Lifestyle 

Recent evidence indicates that intracellular invasion to peculiar epithelial cells may be targeted by neuropeptides. For example, many enteric pathogens, including *S. enterica* serotype Typhimurium, utilize microfold (M)-cells to gain access to the host cell. M-cells represent a small proportion of the specialized follicular-associated epithelium (FAE) overlying mucosa-associated lymphoid tissues in the intestine. Under physiological conditions, M-cells are involved in lumen antigen sampling and transport via transcytosis to lymphocytes and macrophages, thereby generating an immune response [113]. However, these cells are also a highly efficient portal for pathogen entry as they possess many structural attributes relevant to bacterial infection [114,115]. *S. enterica* serotype Typhimurium uses two type-III secretion systems, encoded by genes located within pathogenicity islands to secrete effector proteins SopE, SopB, SipA, and SptP, directly into host epithelial cell including M-cells, where they participate in reorganization of the actin cytoskeleton [116]. Next, using M-cell during invasion, this intestinal pathogen can translocate across the gut epithelium to its deeper layers. Furthermore, to promote intestinal invasion, *Salmonella* is also capable of transforming new FAE epithelial cells into M-cells. Recent evidence indicates that *Salmonella* invasion may be diminished by neuropeptides. This assumption is supported by the fact that CGRP, released from gut-innervating nociceptive sensory neurons, inhibits *Salmonella*-mediated differentiation of M cells. This results in a decreased response to *Salmonella* infection [117]. Whilst a CGRP-dependent reduction in M-cell number seems to contribute to a lower vulnerability to invasion, this is not the sole determinant in decreasing *Salmonella* infection. The influence of CGRP on commensal microbiota involved in the mucosal immunity seems to be equally important. In fact, CGRP has been shown to facilitate the adherence and maintenance of relevant levels of segmental filamentous bacteria (SFB) to Peyer’s patch FAE and villi. These commensal gut residents, through adherence to the epithelium, form a mechanical barrier that is crucial in competing with enteric pathogens [117]. It is also worth adding that SFB commensals affect the priming and induction of Th-17 lymphocytes, which play key roles in mucosal defense not only against enteric bacteria but also against fungal pathogens essentially by induction of antimicrobial peptides production [118]. In conclusion, CGRP released from nociceptor neurons contributes directly and indirectly to defense against invasion of *S. enterica*.

#### 4.5.2. Impact of Neuropeptides on the Obligate Intracellular Lifestyle 

Sometimes to aid entry into a cell, intracellular protozoan pathogens, such as *L. major*, facilitate their engulfment by phagocytic cells. This allows the pathogen to find a safe niche in which to multiply. In such situations, SP- or NPY-mediated inhibition of *Leishmania* ingestion [86,119] can be proposed as defensive action to block intracellular invasion. An antiparasitic effect was also confirmed for neuropeptide PACAP against *Toxoplasma gondii*. *T. gondii* is an obligate intracellular parasite, which infects the small intestine and differentiates to its rapidly replicating stage (tachyzoite), which is able to infect all nucleated cells through active penetration. It has recently been reported that exogenous administration of PACAP in mice leads to decreased systemic parasitic burden as a result of PACAP-mediated phagocytic enhancement by mononuclear cells, contributing to the resolution of the infection [120].

#### 4.5.3. Influence of Neuropeptides on Granulomatous Response

As outlined above, some neuropeptides participate in the pathogenesis of diseases caused by obligate intracellular pathogens, which have evolved to survive and replicate inside host cells after the invasion. To ensure an intracellular lifestyle, several pathogens also participate in granuloma formation. Granulomas are unique evolutionarily ancient structures that contain mainly macrophages and are formed in response to persistent particulate stimuli (infectious or noninfectious), that macrophages alone cannot eradicate. In the case of infectious stimuli, the granulomatous response is associated with bacterial, fungal, and parasitic infection. Currently, the formation of granulomas is understood to contribute to both protective and pathogenic consequences [121]. For example, for *Mycobacterium tuberculosis* early protective granulomas are responsible for eliminating bacteria, but late transmissive granulomas may facilitate bacterial growth and dissemination [122]. The role of neuropeptides in the formation of granulomas involves their immunomodulatory effects on the inflammatory response. The anti-inflammatory activity of α-MSH in an in vitro sarcoidosis-like granuloma model has been documented using peripheral blood mononuclear cells (monocytes and lymphocytes) from sarcoidosis patients challenged with cellular microparticles of *Mycobacterium abscessus*. The granuloma development in this model was reduced in the presence of α-MSH. This was accompanied by a significant decrease in production of pro-inflammatory cytokines and chemokines (comparison to non-treated granuloma controls), including those related to Th1 lymphocytes (IL-2R, IL-7, TNF-α, IFN-γ) and macrophages (CCL2 (MCP-1, CCL3 (MIP-α), CCL4 (MIP-1β), GM-CSF) and others typical of inflammation (IL-6, IL-8, IFN-α) [123]. It has been also shown that SP contributes to granuloma formation, in response to infection by the helminth cestode, *Taenia crassiceps*. Here, SP is involved in induction of proinflammatory cytokines IL-1 and TNF- and Il-6, within the granuloma. These cytokines are recognized to be key mediators of granuloma formation [124]. Likewise, granuloma formation during a normal granulomatous respons, observed in murine schistosomiasis requires the binding of SP to its specific NK1 receptor [125]. Initial granuloma formation by the host in response to agents causing chronic infections is thought to be essential for limiting and eventually clearing infection [124]. However, in some cases, late transmissive granuloma may contribute to the growth and dissemination of intracellular pathogens in the course of tuberculous infection [122,126]. 

## 5. Have Neuropeptides Met Their Expectations as Anti-Infective Agents?

The general principles of using novel peptides as anti-infective therapeutics could involve their use (i) as single agents with direct microbicidal (killing) or microbiostatic (inhibiting growth without killing) action; (ii) in combination with conventional antibiotics or other chemotherapeutics to promote additive or synergistic effects; (iii) as immunostimulatory agents that enhance natural innate or adaptive immunity; and (iv) as endotoxin-neutralizing agents [127,128]. These assumptions at least in part are fulfilled by neuropeptides. Furthermore, one of the beneficial actions that can be attributed to neuropeptides is undoubtedly their indirect effect on commensal microflora [25,129]. The discovery of the antimicrobial properties of neuropeptides placed high hopes for the possibility of their use in the treatment of infectious diseases. Recent examples from clinical trials highlight, however, the duality of action and therefore heterogeneous efficacy of neuropeptides as anti-infective compounds. On the one hand, beneficial health examples of neuropeptide action were reported, including (i) association between high levels of serum SP and lower mortality in severe septic patients [130], or (ii) association between high level of serum CGRP and lower mortality in children with severe pneumoniae [131]. On the other hand, the negative activity of neuropeptides was exemplified by the potential role of SP in severity of diarrhea in cryptosporidiosis in immunocompromised patients [132] or the well-documented detrimental role of SP in periodontal diseases [133]. The complexity of neuropeptide action in clinical trials is yet another example of their duality. After years of experimental research, as well as attempts to use neuropeptides or their analogs in approaches based on animal models, or in clinical trials it seems, however, that the aforementioned hopes have not been fulfilled in many cases. Accordingly, neuropeptides do not currently appear to be highly promising candidates for the treatment of microbial infections. Their therapeutic usefulness appears to be limited mainly because of their rather weak direct antimicrobial action at physiological concentrations and low stability in vivo, connected with the labile nature of peptides. However, it is important to note that many classical AMPs have suffered a similar fate. Currently, there are only a few AMPs in clinical development for the treatment of local skin (bovine indolicidin), nasal (synthetic antimicrobial peptidomimetic Lytixar (LTX-109)), or oral (LL-37) bacterial infections, all of which are intended for topical use at the site of infection. The only AMP in clinical trials for intravenous administration is human-derived Lactoferrin 1–11 (hLF1–11) for the treatment of life-threatening infections in patients after stem cell transplantation [134]. 

Regarding peptides derived strictly from the neuroendocrine system, to date, only two peptides, namely an α-MSH derivative and ghrelin have entered Phase II clinical trials for the treatment of vulvovaginal candidiasis and chronic respiratory infection, respectively. Encouragingly, positive anti-candidal efficacy has been reported for the α-MSH derivative and anti-inflammatory efficacy has been reported for both compounds [135].

Neuropeptides as naturally occurring protein molecules can be influenced by many factors in the host environment. In this aspect, both intrinsic factors (e.g., post-translational modifications) and acquired ones (e.g., pathogens and their released proteases) can potentially lead to disturbance or perturbation or modulation of neuropeptides activity. Some of these factors are discussed below.

### 5.1. Glycosylation of Neuropeptides and Their Receptors

Glycosylation, the enzymatic addition of monosaccharide or glycan, is the most common and complex post-translational modification of the polypeptide chain. Glycosylation adds increased structural diversity to proteins and peptides, impacting on their conformation and physicochemical properties [136,137,138] and in turn translating to their biological activities [139,140]. Changes in glycosylation profile are known to be associated with various physiological and pathological conditions and can modulate the biological recognition events, including inflammatory responses and host–microbe interactions [140,141,142]. Both linked glycans and free oligosaccharides are also a key factor in shaping the microbiome [143,144]. 

Recent studies have shown that the presence of glycans impacts the biological activity of neuropeptides. The neurokinin 1 receptor (NK1R) is a G protein-coupled receptor for SP, with two putative N-linked glycosylation sites, Asn^14^ and Asn^18^. This sugar moiety has been shown to be critical for receptor function, since ligand binding to the unglycosylated form of NK1R demonstrated reduced SP-induced IL-8 secretion [145]. Likewise, the double mutant Asn^18^Thr/Asn^31^Thr lacking both glycosylation sites for sst3 subtype of somatostatin receptor showed a significant reduction in high affinity binding of somatostatin [146]. The glycosylation of neuropeptide receptors therefore appears to have functional consequences on the biology of their ligands. Likewise, the importance of peptide glycosylation is demonstrated by the fact that one-third of the 279 classified peptide hormones carry O-glycans. Many of the well-characterized neuropeptides, such as NPY, VIP, CGRP, galanin, and secretin are glycosylated [147]. For example, the glucagon family members including VIP share a highly conserved O-glycan at Thr^7^, while the NPY family members share a conserved O-glycan at Thr^32^. Taking advantage of this, three glycovariants of VIP and NPY were chemoenzymatically synthesized to contain the most common O-GalNAc-type structures namely Tn (GalNAcα1-O-Ser/Thr), T (Galβ1-3GalNAcα1-O-Ser/Thr), and sialylated ST (NeuAcα2-3Galβ1-3GalNAcα1-O-Ser/Thr) at NPY Thr^32^ and VIP Thr^7^ to explore how O-glycans on these neuropeptides modulate receptor activation. Madsen et al. showed that: (i) O-linked VIP and NPY dampened their cognate receptor activation which was found to correlate positively with the size of the attached O-glycans, (ii) O-glycans located in the receptor-binding domain of the NPY had a greater impact on receptor activation than glycans in the receptor-binding domain of VIP, indicating that the nature of the entire peptide is also important [147]. Furthermore, the presence of O-glycans attached to atrial natriuretic peptide (ANP) attenuates its acute renal and cardiovascular actions in vivo, whereas in vitro O-glycosylation was shown to protect ANP from proteolytic cleavage and modulate interactions with its cognate receptor [148]. Recently, it has also been demonstrated that O-glycans within the receptor-binding motifs of members of the NPY and glucagon families substantially extend peptide half-life and modulate receptor activation properties [147]. Many of the identified O-glycosites are conserved and are predicted to serve roles in proprotein processing, receptor interaction, biodistribution, and biostability [Madsen, 2020]. For this reason, synthetic glycosylation via chemoenzymatic addition of N- or/and O-glycans may be an effective and promising strategy to enhance the bioavailability, half-life, and expected biological effects of therapeutic peptides [149,150,151].

### 5.2. Vulnerability of Neuropeptides to Host and Bacterial Proteases 

**Figure 2:**
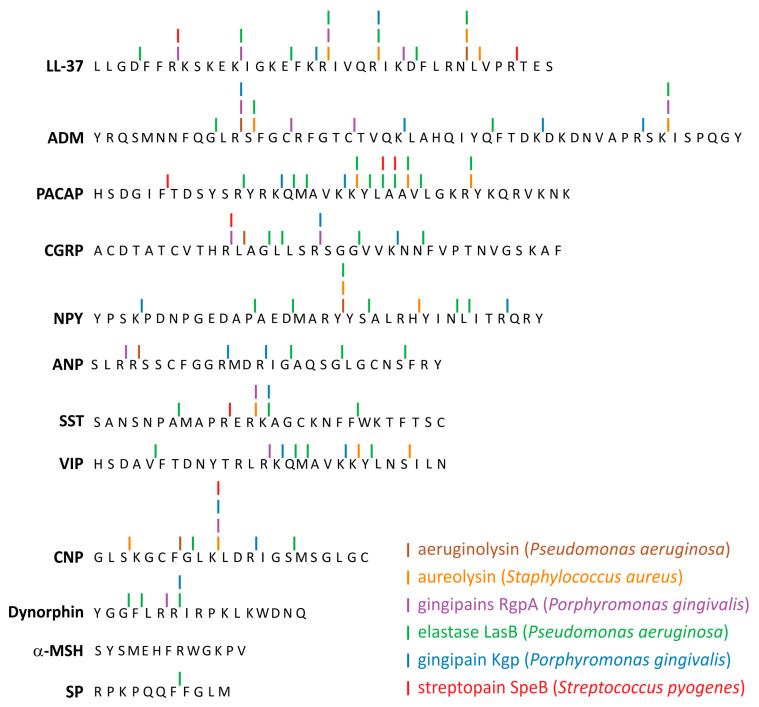
Potential cleavage sites in neuropeptide sequences subjected to selected bacterial proteases. The sites were identified using PROSITE scanning program and cleavage patterns created based on data available in MEROPS database for the individual peptidases. In the pattern, we considered three positions before and after the cleavage site, i.e., P3 P2 P1 P1’ P2’ P3’. Antimicrobial peptide LL-37 was shown for comparison.

Neuropeptides as others endogenous peptides can interact with various endogenous peptidases. It is important to note that levels of these proteins and enzymes can be regulated/altered by particular disease. At sites of infection in vivo, in patients suffering from periodontitis, specific CGRP degradation by carboxypeptidase present in the extracellular gingival milieu has been reported [152]. Similarly, peptide degradation by the classical AMP, cathelicidin LL-37 has also been reported in the same sites of infection [153], supporting the major role of proteases, including bacterial proteases [153] in resistance to the actions of antimicrobial peptides Although there are no studies on the mechanisms of neuropeptide resistance in microorganisms, by analogy with classical mammals AMPs such as LL-37 and defensins, it is assumed that they are similar. The induction of intrinsic adaptive resistance to AMPs in bacteria is tightly regulated in response to environmental pressure. This response includes: (i) the reduction of bacterial negative cell-surface charge through the incorporation of positively charged molecules into the cell membrane, (ii) efflux pumps to expel AMPs, (iii) proteolytic degradation of AMPs by microbial exotoxins/proteases, (iv) induction of AMP trapping mechanisms [134,154]. When comparing neuropeptides with AMPs in this respect, it should be borne in mind that due to the very low physiological concentration of neuropeptides in mammals, the emergence of selection pressure appears less likely. Proteolytic degradation of neuropeptides by microbial proteases, however, seems to be a different and more likely matter. The findings that various bacteria such as *P. aeruginosa*, *E. faecalis*, *Proteus mirabilis*, *S. enterica*, *S. pyogenes*, *Burkholderia cenocepacia*, *Vibrio cholera* and pathogenic yeasts such as *C. albicans* can cleave pivotal antimicrobial peptides including LL-37 appear to support this notion [15,16,17,18,19,20,21,22,23,24,25,26,27,28,29,30,31,32,33,34,35,36,37,38,39,40,41,42,43,44,45,46,47,48,49,50,51,52,53,54,55,56,57,58,59,60,61,62,63,64,65,66,67,68,69,70,71,72,73,74,75,76,77,78,79,80,81,82,83,84,85,86,87,88,89,90,91,92,93,94,95,96,97,98,99,100,101,102,103,104,105,106,107,108,109,110,111,112,113,114,115,116,117,118,119,120,121,122,123,124,125,126,127,128,129,130,131,132,133,134,135,136,137,138,139,140,141,142,143,144,145,146,147,148,149,150,151,152,153,154,155,156,157,158,159]. Since many of the bacteria discussed produce exotoxins or proteases, we performed bioinformatic analyses to assess the potential sensitivity of the neuropeptides discussed in this paper to these enzymes (Appendix A, Figure 2). We considered the action of 12 proteolytic enzymes from 6 pathogenic bacteria (*P. aeruginosa*, *P. gingivalis*, *S. typhimurium*, *S. aureus*, *S. pyogenes*, *L. longbeachae*) on 11 neuropeptides. The cleavage sites were identified using PROSITE scanning program [160] and the cleavage patterns were created based on data available in MEROPS database for the individual peptidase [161]. The enzymes were chosen because they cleave the classical AMP, LL-37 and/or pivotal human interleukins [162]. The vast majority of neuropeptides seemed to be susceptible to the 6 out of 12 proteases studied using the in silico approach. CNP could be cleaved by six proteases, ADM, CGRP, and SST by five proteases, ANP, NPY, PACAP, and VIP by four, Dynorphin by three, whereas SP by one. α-MSH did not reveal cleavage sites recognized by any of the enzymes studied. For comparison, LL-37 was subject to the same in silico analysis. These results indicate that pathogenic bacteria can cleave many neuropeptides and as a result modulate the action of these compounds. 

### 5.3. Duality of Action in Murine Models

Evidence for the dual role of neuropeptides in preclinical interventions in murine models of infectious diseases are summarized in Table 2 and Table 3. They involve (i) recent approaches for the use of exogenous neuropeptides as anti-infective drugs in murine models of diverse infections (Table 2); (ii) animal models of infectious disease aimed at modulating the expression of endogenous neuropeptides at the site of infection to boost innate mechanisms of immune response (Table 3). The two most commonly used neuropeptides in these studies were SP and VIP. Considering the results presented in Table 2 and Table 3 in more detail, striking similarities can be seen in the ineffectiveness of SP, regardless of the type of infectious disease or etiological factor or pathogen involved. This unexpected observation seems to be contradictory to common expectations. The duality of action of this neuropeptide is reflected by the fact that in some conditions, such as salmonellosis, healing was achieved. In contrast, in other disorders such as infectious keratitis, this neuropeptide has a completely different effect leading to worsening and disease exacerbation. Similar observations come from studies focused on endogenous expression of SP. In this case, in the course of various meningitis, irrespective of the bacterial pathogen involved, increased SP expression was associated with unsatisfactory clinical outcome. Alternatively, increase in SP during pneumonia corresponded with beneficial clinical parameters. 

In contrast to SP, administration of VIP has been shown to act beneficially in the course of the variety of infectious disorders, mainly by extinguishing inflammatory reactions. It essentially occurred through decrease in inflammatory cytokines and adhesion molecules of inflammatory cells as well as increase in growth factors for cells involved in tissue repair. Interestingly, VIP achieved healing in corneal infection models, and beneficial results in periodontal infection models and infectious keratitis model, and indeed was successful in all models studied, reflecting its well-recognized anti-inflammatory action. The immunosuppressive potential of neuropeptides such as VIP and PACAP could probably be exploited in future to restore homeostasis. These so-called anti-inflammatory neuropeptides could contribute directly to the later stages of inflammation, that require a dampening of the inflammatory response, in advance of healing and repair.

## 6. Conclusion and Future Prospects

In conclusion, the pleiotropic nature of neuropeptides indicates that they have pro-infective and anti-infective effects, as well as virulence-enhancing and virulence-attenuating properties. By having such a pleiotropic nature, which is largely dependent on specific virulence traits of pathogens, it is impossible to classify neuropeptides as compounds with a uniformly defined pattern of action. Considering their anti-infective benefits in vitro, neuropeptides have been shown to attenuate common stages of microbial pathogenesis such as adhesion, invasion, intracellular lifestyle. Nevertheless, these positive effects do not appear to be mirrored in preclinical interventions in murine in vivo models or in the relatively few clinical trials completed to date.

Evidence to date would suggest that the anti-infective activities of the majority of neuropeptides appear not to be as pronounced as their antibiotic counterparts, indicating that this aspect of their therapeutic effectiveness remains elusive. A notable exception is, however, VIP which has proved to be successful in murine models of certain infectious diseases. 

In summary, although neuropeptides, as important components of innate immunity, are unlikely to be universally recognized as therapeutic antimicrobial agents, their value as natural factors involved in modulating host–pathogen interactions is currently being elucidated. In this respect, physiologically functional neuropeptides are important host constituents that contribute to general health by maintaining the balance between immunity to pathogens and their tolerance. Therefore, we are faced with the task of increasing research efforts in this field to fully recognize the immunomodulatory properties of neuropeptides as additional players in maintaining homeostasis. 

Furthermore, the findings that neuropeptides have numerous anti-infective strategies but also virulence-enhancing properties raises future questions regarding (i) how microorganisms sense neuropeptides; (ii) how neuropeptides induce microbial virulence; (iii) whether bacteria can subvert action of neuropeptides. All these issues should be addressed in the future.

## Figures and Tables

**Table 2:** Exogenous application of neuropeptides as anti-infective drugs in murine models of diverse infections.

*NP	Condition	The Site of Administration of Exogenous NP	Effect/Evaluated Parameter	Clinical Outcome	Pathogen (Virulence Factor)	References
SP	salmonellosis	intraperitoneal injection	IFN-γ, IL-12p40 ↑	reduced susceptibility to salmonellosis	*Salmonella*	[163]
SP	infectious keratitis	intraperitonel injection	HGF, FGF-7 ↑ IL1-β, MIP-2, TNF-α ↑ Bcl-2 ↑ TGF-β ↓	enhancing the inflammatory response	*Pseudomonas aeruginosa*	[164]
SP	infectious keratitis	intraperitoneal injection	neutrophil infiltration ↑ Bacterial upload ↑ mRNA of NFκB, IFN-γ, TNF-α, MIP-2, IL-18, IL-6 ↑ IL-1β ↑ VIP, TGF – β, IL-10 ↓	exacerbated disease scores	*Pseudomonas aeruginosa*	[165]
VIP	cornea infection	intraperitoneal injection	ICAM-1, VCAM-1 ↓ LFA-1, VLA-4 ↓ cellular infiltration ↓	better disease outcome	*Pseudomonas aeruginosa*	[166]
VIP	infectious keratitis	intraperitoneal injection	EGF, FGF, HGF, VEGF-A ↑ angiogenesis ↑	healing promotion and restoration of tissue homeostasis	*Pseudomonas aeruginosa*	[167]
VIP	periodontitis	intraperitoneal injection	sRANKL ↓ OPG ↓ osteoblastic density ↑	downregulation of the inflammatory response and inhibition of alveolar bone loss	*Escherichia coli (LPS)*	[168]
VIP	bacterial keratitis	intraperitoneal injection	corneal perforation ↓ bacteria number ↓ MPO ↓ IL-1β, TNF-α, MIP-2 ↓ TGF-β, IL-10, SOCS3, COX-2, ALOX12 ↑	amelioration of the disease pathogenesis	*Pseudomonas aeruginosa*	[169]
PACAP	subacute ileitis-associated human gut microbiota	intraperitoneal injection	proinflammatory cytokines in CNS: TNF-α, IL-6 ↓ number of T- cell in large intestinal tract ↓ changes in intestinal microbiota mRNA: lactobacilli ↑	alleviation of subacute ileitis	*Toxoplasma gondii* as inductor of ileitis after fecal microbiota transplantation	[170]

*NP—neuropeptide; other acronyms are in the list of abbreviations; **↑**—increase, **↓**—decrease.

**Table 3:** Endogenous expression of neuropeptides at the site of diverse infections in murine models.

*NP	Condition	The Source of Endogenous NP	Effect/Evaluated Parameter	Clinical Outcome	Pathogen (Virulence Factor)	References
SP	meningitis	whole brain homogenates and glial cells	blood-brain barrier permeability ↑ cytokines in CNS: TNF-α, IL-6 ↑ IL-10 ↓	initiation and progression of neuroinflammation	*Streptococcus pneumoniae*	[171]
SP	infectious keratitis	cornea	IL1-β, MIP-2, TNF-α, IL-6, IL-18, IFN-gamma ↑ NF-κβ activation↑, IL-10 ↓	augmenting inflammation in the cornea after infection	*Pseudomonas aeruginosa*	[172]
SP	periodontitis	gingival fibroblasts	HIF-1α and the RANKL/OPG ratio ↑	participation in periodontitis	*Porphyromonas gingivalis*	[173]
SP	cornea infection	cornea	IFN-gamma ↑ IL-18 ↑	induction of the immune response	*Pseudomonas aeruginosa*	[174]
SP	pneumonia	bronchoalveolar lavage fluid and plasma	pulmonary neutrophils recruitment ↑ phagocytosis of pathogen ↑	increased survival and enhanced immune response to the infection	*Pseudomonas aeruginosa*	[175]
SP	meningitis	effect observed in WT mouse and compared to NK-1 knockout mouse without SP signalling	TNF-α, IL-6 ↑ IL-10 ↓	initiation and/or progression of CNS inflammation	*Borrelia burgdorferi*	[176]
SP	meningitis	effect observed in WT mouse and compared to NK-1 knockout mouse without SP signalling	cellularity ↑ astrogliosis ↑ demyelination ↑ TNF-α, IL-6 ↑ IL-10 ↓	initiation and/or progression of CNS inflammation	*Neisseria meningitidis*	[176]
CRH	ileal inflammation	ileal loops lysates	SP level ↑ ileal secretion, epithelial cells damage ↑ neutrophil infiltration, ileal MPO activity ↑	acute intestinal inflammatory response	*Clostridium difficile* (toxin A)	[177]
CRH and Ucn	antibiotic-associated colitis	ileal loops lysates	IL-8, monocyte chemoattractant protein – 1 ↑ intestinal secretion, epithelial cells damage, histological scoring of inflammation ↑	inflammatory response in the intestine	*Clostridium difficile* (toxin A)	[178]
CGRP	keratitis	cornea	macrophages aggregation ↑ in vitro test on isolated mouse macrophages with exogenous CGRP: IL-10 ↑, Il-1β, TNF-α, IL-6 ↓	protective role against inflammation in *A. fumigatus* keratitis	*Aspergillus fumigatus*	[179]

*NP—neuropeptide; other acronyms are in the list of abbreviations; **↑**—increase, **↓**—decrease.

## Data Availability

The bioinformatics data presented in this study are available on request from the corresponding author. The data are not publicly available.

## Abbreviations

ADM	Adrenomedullin
ALOX12	Arachidonate 12-Lipoxygenase
AMPs	Antimicrobial Peptides
ANP	Atrial Natriuretic Peptide
Bcl-2	B-cell Lymphoma 2
CGRP	Calcitonin Gene-Related Peptide
CNP	C-type Natriuretic Peptide
COX-2	Cyclooxygenase-2
CRH	Corticotropin Releasing Hormone
Ef-Tu	Ribosomal Elongation Factor Thermo-Unstable
EGF	Epidermal Growth Factor
FGF	Fibroblast Growth Factors
GAPDH	Glyceraldehyde 3-Phosphate Dehydrogenase
GPCR	G Protein- Coupled Receptors
HGF	Hepatocyte Growth Factor
HHQ	4-hydroxy-2-heptylquinoline
HIF-1α	Hypoxia-inducible factor 1 α
Hlf1–11	Human-Derived Lactoferrin 1–11
ICAM-1	Intercellular Adhesion Molecule 1
IFN-γ	Interferon-γ
IsaB	Immunodominant Staphylococcal Antigen B
LFA-1	Lymphocyte Function-Associated Antigen 1
Lkt	Leukotoxin
MIP-2	Macrophage Inflammatory Protein 2
MPO	Myeloperoxidase
NFκB	Nuclear Factor Kappa-Light-Chain-Enhancer of Activated B Cells
NPY	Neuropeptide Y
OPG	Osteoprotegerin
PACAP	Pituitary Andenylate Cyclase-Activating Polypetide
PA-I	Lectin/Adhesin PA-I
pavA	Pneumococcal Adherence and Virulence Factor A
PNS	Peripheral Nervous System
PQS	2-heptyl-3,4-dihydroxyquinoline
PRRs	Pattern Recognition Receptors
PtxR	HTH-Type Transcriptional Regulator PtxR
QS	Quorum Sensing
SEC2	Enterotoxin C2
SFB	Segmental Filamentous Bacteria
SOCS3	Suppressor of Cytokine Signaling 3
SP	Substance P
sRANKL	Soluble Receptor Activator of Nuclear Factor-κB Ligand
SST	Somatostatin
TGF-β	Transforming Growth Factor β
TNF-α	Tumor Necrosis Factor α
TRPV1	Transient Receptor Potential Vanilloid 1
Ucn	Urocortins
VCAM-1	Vascular Cell Adhesion Molecule 1
VEGF-A	Vascular Endothelial Growth Factor A
VIP	Vasoactive Intestinal Peptide
VLA-4	Very Late Antigen-4
α-MSH	α-Melanocyte Stimulating Hormone

## Appendix A

The number of potential cleavage sites in neuropeptides subjected to selected proteases. The sites were identified using PROSITE scanning program and cleavage patterns created based on data available in MEROPS database for the individual peptidases. In the pattern, we considered three positions before and after the cleavage site, i.e., P3 P2 P1 P1’ P2’ P3’. Antimicrobial peptide LL-37 was included for comparison.

**Name of Protease**	**Species**	**Accession Number**	**ADM**	**α-MSH**	**ANP**	**CGRP**	**CNP**	**Dynorphin**	**NPY**	**PACAP**	**SST**	**SP**	**VIP**	**LL-37**
Alkaline protease aeruginolysin	*Pseudomonas aeruginosa*	APRA_PSEAE	1		1	1	1		1					1
Arg specific gingipains RgpA	*Porphyromonas gingivalis*	CPG1_PORGN	4		1	2	1	1			1		1	4
Arg specific gingipains RgpB	*Porphyromonas gingivalis*	CPG2_PORGI												
Aureolysin	*Staphylococcus aureus*	AURE_STAAU	2				2		2	3	1		2	4
C5a peptidase (ScpA)	*Streptococcus pyogenes*	C5AP_STRP6												
Elastase (LasB, pseudolysin)	*Pseudomonas aeruginosa*	ELAS_PSEAE	4		3	4	2	3	6	10	3	1	4	7
Gingipains [Lys-specific gingipain (Kgp)	*Porphyromonas gingivalis*	KGP83_PORGN	4		2	2	2	1	2	2	1		2	2
LasA protease staphylolysin	*Pseudomonas aeruginosa*	LASA_PSEAE												
MspA	*Legionella longbeachae*	MSPA_LEGLO												
PgtE (OmpE, protein E)	*Salmonella Typhimurium*	PGTE_SALTY												
SlyCEP (ScpC)	*Streptococcus pyogenes*	Q3HV58_STRPY												
Streptopain (SpeB)	*Streptococcus pyogenes*	SPEB_STRPY				1	1			3	1			2
